# Thermo-Mechanical Phase-Field Modeling of Fracture in High-Burnup UO_2_ Fuels Under Transient Conditions

**DOI:** 10.3390/ma18051162

**Published:** 2025-03-05

**Authors:** Merve Gencturk, Nicholas Faulkner, Karim Ahmed

**Affiliations:** 1 Department of Nuclear Engineering, Texas A&M University, College Station, TX 77843, USA; mgencturk@tamu.edu (M.G.);; 2 Department of Materials Science and Engineering, Texas A&M University, College Station, TX 77843, USA

**Keywords:** uranium dioxide, high burnup, fracture, phase field

## Abstract

This study presents a novel multiphysics phase-field fracture model to analyze high-burnup uranium dioxide (UO_2_) fuel behavior under transient reactor conditions. Fracture is treated as a stochastic phase transition, which inherently accounts for the random microstructural effects that lead to variations in the value of fracture strength. Moreover, the model takes into consideration the effects of temperature and burnup on thermal conductivity. Therefore, the model is able to predict crack initiation, propagation, and complex morphologies in response to thermal gradients and stress distributions. Several simulations were conducted to investigate the effects of operational and transient conditions on fracture behavior and the resulting cracking patterns. High-burnup fuels exhibit reduced thermal conductivity, elevating temperature gradients and resulting in extensive radial and circumferential cracks. Transient heating rates and temperatures significantly affect fracture patterns, with higher heating rates generating steeper gradients and more irregular crack trajectories. This approach provides critical insights into fuel integrity during accident scenarios and supports the safety evaluation of extended burnup limits.

## 1. Introduction

Nuclear fuel vendors are seeking to raise burnup limits for light water reactor (LWR) fuel in U.S. commercial power reactors to enhance efficiency and reduce spent fuel generation. However, the Nuclear Regulatory Commission (NRC) will likely require nuclear power plants (NPPs) to analyze a number of design-basis accidents (DBAs) and their potential consequences before such an extension can be approved. In the pursuit of extending burnup to rod-average levels exceeding the current regulatory limit of 62 GWd/tU, it is crucial to evaluate design limits while identifying potential new damage mechanisms specific to this higher burnup and assessing burnup-related degradation phenomena [1].

The performance of light water reactors (LWRs) is significantly affected by the accumulation of fission products within the fuel. These fission products, generated during nuclear reactions, include noble gases such as xenon and krypton. Due to their low solubility in UO_2_, these gases migrate through the matrix toward grain boundaries and pores, leading to the formation of intra- and inter-granular bubbles. The presence of these bubbles causes swelling and reduces the thermal conductivity of the fuel pellets, resulting in a pronounced temperature gradient, and once an interconnected network of open porosity forms, the gases escape into the fuel-cladding gap and plenum. This process, known as fission gas release (FGR), poses a potential limitation on the operational lifespan of nuclear fuel rods, as it increases internal gas pressure and may ultimately compromise the integrity of the fuel cladding [2].

The neutron energy spectrum in light water thermal reactors (LWRs) facilitates the production of fissile 239-Pu through resonance neutron absorption by 238-U, predominantly in the periphery of the UO_2_ fuel pellet. Consequently, the local burnup near the fuel pellet edge surpasses the average pellet burnup, triggering a restructuring process that results in the emergence of a new fuel microstructure. In high-burnup UO_2_ fuel, these changes are marked by the formation of small bubbles and the fragmentation of grains near the pellet’s outer region. As early as 1962, Bleiberg et al. [3] demonstrated a correlation between the release of a fraction of theoretically produced fission gases from fuel under in-pile conditions and factors such as fuel burnup and operating temperature. They also documented significant changes in the microstructure in these fuels. This modified configuration was subsequently termed the high-burnup structure (HBS) in the work of [4] and is characterized by recrystallization accompanied by the formation of grains or sub-grains. Some researchers referred to it as the high-burnup rim structure, emphasizing its initial observation at the radial periphery of the LWR fuel pellet [5,6,7,8,9].

The interest in increasing burnup led to investigations into high-burnup effects. Results from the High-Burnup Effects Program (HBEP) indicated that the HBS was identified as a potential contributor to increased fission gas release observed with high burnup, although the precise quantification of gas release from the HBS was not achieved [10]. These findings initiated investigations into fuel behavior due to concerns about fuel integrity under transient conditions. As a result, fuel behavior during loss-of-coolant accidents (LOCAs) has been extensively studied over the past decades.

Recent LOCA tests performed in Halden and Studsvik have renewed interest in this field. Test results indicate that high-burnup fuel pellets may pulverize into extremely fine fragments. Test results suggest that an increase in burnup elevates the likelihood of fuel fragmentation, relocation, and dispersal (FFRD) during an LOCA. High-burnup fuel pellets, in particular, may pulverize into extremely fine fragments. It was reported that a significant fraction of these particles is fine enough to relocate axially and be ejected through the rupture opening into the coolant [11,12,13].

Thermal stresses induced by the first power ramp to nominal reactor power result in the formation of multiple radial macro-cracks within the fuel pellets. The radial and axial temperature gradients are affected by burnup as the pellet’s thermal conductivity degrades. Thermal conductivity degradation is one of the most significant consequences of the HBS. As burnup increases, it significantly impacts the accumulation of fission products, radiation-induced damage, and density, collectively leading to decline in the thermal conductivity of UO_2_. This reduction elevates the central temperature of the fuel. Bagger et al. [14] reported a degradation in the thermal conductivity of fuel with increasing burnup. They observed that the thermal conductivity of the fine-grained porous rim in high-burnup fuel was significantly lower than that of as-fabricated UO_2_. Factors such as reduced grain size, increased porosity, and accumulated fission gases contributed to the formation of thermal barriers. Also, Nakamura et al. [15] reported that the thermal diffusivity of high-burnup UO_2_ at room temperature was less than half that of as-fabricated UO_2_. This reduction was primarily attributed to fission products, density reductions, and radiation-induced damage, including point defects and defect clusters, with potential contributions from the presence of fission gas bubbles [16,17].

Since the first observation of HBS, numerous studies have been conducted employing modern electron microscopy tools in experimental investigations [18,19,20]. However, experimental techniques to study HBS have been limited due to the challenges associated with highly irradiated fuel. This underscores the need for advanced modeling approaches that incorporate microstructure and irradiation effects to enhance the predictive accuracy of fuel behavior under transient scenarios.

In this work, a phase-field model is developed to treat fracture as a phase transition, incorporating thermo-mechanical coupling to analyze temperature-dependent fracture patterns in UO_2_ fuel pellets under transient reactor conditions. The objective of this study is to assess the effects of transient conditions on UO_2_ fuel as a function of temperature, heat rate, and burnup to investigate the resulting fracture patterns. While two previous studies employed a multiphysics phase-field fracture model to investigate UO_2_ fracture [21,22], such a method has not yet been applied to the study of high-burnup UO_2_ fuel under accident-like transient conditions. Moreover, the treatment of the process of fracture in the phase-field approach presented here as a phase transition is novel and enables the incorporation of stochastic effects, which is instrumental for including the underlying microstructure and defects that eventually manifest themselves into distinct cracking patterns and/or the measured fracture strength.

## 2. Methodology

### 2.1. Multiphysics Phase-Field Modeling of Thermo-Mechanical Fracture as Phase Transition

This section aims to develop a phase-field model that treats fracture as a phase transition, incorporating thermo-mechanical coupling to evaluate its effects on UO_2_ fuel pellets during transient reactor conditions. First, the formulation of the phase-field fracture model will be presented. The stochastic terms employed to represent the random nature of fracture will be discussed. Next, the governing equations for mechanical coupling will be presented. Lastly, the heat equation and the choice of thermal conductivity correlation as a function of burnup and temperature will be introduced. All the UO_2_ physical properties and corresponding model parameters utilized in our simulations are also listed.

#### 2.1.1. Phase-Field Modeling of Fracture

Thermal expansion leads to an increase in the total strain of a system and results in a rise in elastic strain energy. When the energy reaches a critical point, it becomes energetically favorable to nucleate cracks in order to reduce the total free energy [21,22,23]. Fracture modeling within the phase-field approach accommodates states of undamaged, partially damaged, and fully damaged material, with the thermodynamic potential offering detailed insights into these conditions while capturing the evolution of the cracks. Within this methodology, a crack is characterized by the phase-field variable η, which varies continuously from 0 to 1 to minimize the system’s total free energy. A value of η=1 corresponds to a fully damaged state (e.g., a crack), whereas η=0 represents an undamaged material state. The Allen–Cahn equation [23] is utilized to evolve the phase-field parameter η and it takes the following form:

(1) $$\begin{array}{c} \frac{\partial \eta }{\partial t}=-L\;\;\left(\frac{\delta \Psi_{total}}{\delta \eta }\;\right) \end{array}$$

where *L* is the order parameter mobility for η and $\Psi_{total}$ is total free energy density. To account for microstructural variations, a stochastic term, λ, is added to the Allen–Cahn equation:

(2) $$\begin{array}{c} \frac{\partial \eta }{\partial t}=-L\;\;\left(\frac{\delta \Psi_{total}}{\delta \eta }\;+\lambda \right) \end{array}$$

Note that in classical phase transition models, such as solidification or precipitation, stochastic noise terms are typically associated with thermal fluctuations in atoms at a specific temperature. However, the stochastic term in Equation (2) in this context does not represent thermal fluctuations but rather accounts for the underlying randomness of microstructural features and/or defects that are not directly considered in the model such as pores, grain boundaries, precipitates, and dislocations. Moreover, even if those features are directly accounted for, as in some micromechanical models, this stochastic term should still be used to incorporate the randomness in the size, morphology, and spatial distribution of those features or defects. In general, the magnitude of the stochastic term should be carefully selected to ensure that it reflects the system’s physical behavior. In classical thermal fluctuation models, the fluctuation–dissipation theorem is used to relate the magnitude of fluctuations to the system’s temperature and dissipation properties. In this work, the magnitude of the stochastic term is empirically tuned to introduce a small fluctuation of only 1% in the strain energy, which is a reasonable assumption given the lack of an exact theoretical treatment. The free energy density is defined as

(3) $$\begin{array}{cc} \; & \Psi_{total}=\;\Psi_{elastic}+\;\Psi_{geometrical\;crack\;energy}+\;\Psi_{gradient} \end{array}$$

where $\Psi_{elastic}$, $\Psi_{geometrical\;crack\;energy}$, and $\Psi_{gradient}$ are the elastic, geometrical crack, and gradient contributions to the energy, respectively. The elastic energy is accepted as

(4) $$\begin{array}{c} \Psi_{elastic}=\frac{1}{2}\epsilon :E:\epsilon \end{array}$$

ϵ is the strain tensor and E is the elastic modulus of the material. The representation of the elastic modulus will be discussed in detail later. The gradient energy is

(5) $$\begin{array}{c} \Psi_{gradient}=\frac{k}{2}{\left|\eta \right|}^{2} \end{array}$$

where *k* is the gradient energy coefficient. In line with most phase-field fracture models, we incorporate cohesion energy into geometrical crack energy, considering cohesion energy and double-well potential, as follows:

(6) $$\begin{array}{c} \Psi_{geometrical\;crack\;energy}=Af\left(\eta \right)+Bw\left(\eta \right) \end{array}$$

where *A* represents the cohesion contribution and *B* represents the double-well contribution. Levitas et al. [24] defined energy associated with damage as cohesion energy. Cohesion energy is expressed as follows:

(7) $$\begin{array}{c} \Psi_{cohesion}=Af\left(\eta \right) \end{array}$$

where f(η) represents the cohesion energy function. w(η) represents the double-well potential function, as described in [23], and is given by

(8) $$\begin{array}{c} w\left(\eta \right)=\eta^{2}{\left(1-\eta \right)}^{2} \end{array}$$

w(η) serves as an energy barrier between the undamaged and fully damaged states, introducing additional irreversibility constraints to prevent crack healing. The mathematical form of the cohesion energy is defined next.

In the fully damaged state (η=1), the parameter *A* represents the maximum cohesion energy, expressed as f(1)=1. Elastic stresses and energy are entirely released in this state, leading to I(1)=0. The I(η) function acts as an interpolation/degradation function between the damage-free and fully damaged states. In the damage-free state (η=0), the only energy present is elastic energy, characterized by f(0)=0 and I(0)=1. Based on the key requirements defined above, this work adopts the following formulas for cohesion energy and interpolation/degradation functions:

(9) $$\begin{array}{c} f\left(\eta \right)=\frac{\eta^{2}}{\eta^{2}+{\left(1-\eta \right)}^{2}} \end{array}$$

(10) $$\begin{array}{c} I\left(\eta \right)=1-f\left(\eta \right)=\frac{{\left(1-\eta \right)}^{2}}{\eta^{2}+{\left(1-\eta \right)}^{2}} \end{array}$$

Consequently, the model parameters *A* and *B* (recall Equation (6)) allow independent control over fracture energy and surface energy. Hence, this formulation of the phase-field fracture model puts it on equal footing with traditional phase-field models for classical phase transitions, as outlined by [24,25]. To identify the intrinsic surface energy, the two phases (here, the intact solid and crack) must be able to coexist. This only happens when the stored strain energy in the solid phase is equal to the cohesion energy, which represents the energy cost of breaking the atomic bonds of the initially solid phase to create the crack. The difference in the free energy between the two phases under such conditions stems only from the double-well contribution w(η) and is non-zero only in the interfacial area, e.g., at the crack surface. The equilibrium solution of the Allen–Cahn equation for the double-well potential is known analytically and the surface energy and interface/crack width are given in terms of B and the gradient energy coefficient (recall Equation (5)). Therefore, the parameter B is obtained as a function of the length scale and surface energy as follows:

(11) $$B=\frac{12\gamma }{\ell }$$

The surface energy, γ, is given by $\gamma =\frac{\sqrt{Bk}}{3\sqrt{2}}$, and the length scale (interface width), *l*, is $l=2\sqrt{2}\sqrt{\frac{k}{B}}$. For ideal brittle fracture, the critical energy release rate is a factor of two of the surface energy, which equals 2γ. The stress, σ, is defined as

(12) $$\begin{array}{c} \sigma =\frac{\partial \Psi_{elastic}}{\partial \epsilon } \end{array}$$

The work of fracture is equal to the area under the stress–strain curve and expressed as

(13) $$\begin{array}{c} \int_{0}^{\epsilon_{c}}\sigma :d\epsilon =\Psi_{elastic}=\frac{1}{2}E\epsilon^{2}=\frac{\sigma^{2}}{2E} \end{array}$$

where $\epsilon_{c}$ is the critical strain at η=1 (fully damaged state). In the same context, the critical stress can be expressed as

(14) $$\begin{array}{c} \sigma_{c}=\sqrt{2E\Psi_{elastic}} \end{array}$$

According to the second law of thermodynamics, for a phase to be considered unstable, the second derivative of the free energy with respect to the order parameter must be negative. This relationship arises because a negative second derivative indicates that small perturbations in the system will lead to a decrease in free energy, thereby driving the system away from that phase and indicating thermodynamic instability. Therefore, the criteria for instability can be expressed as

(15) $$\begin{array}{c} \frac{\partial^{2}\psi }{\partial \eta^{2}}\leq 0 \end{array}$$

This condition is satisfied when $\Psi_{elastic}=A+B$ and the critical stress can be expressed as follows:

(16) $$\begin{array}{c} \sigma_{c}=\sqrt{2E\Psi_{elastic}}=\sqrt{2E(A+B)} \end{array}$$

Substituting Equation (11) into Equation (16), we obtain

(17) $$\begin{array}{c} \sigma_{c}=\sqrt{2E(A+\frac{12\gamma }{\ell })} \end{array}$$

In Griffith’s theory of brittle fracture [26], the critical stress $\sigma_{c}$ is proportional to the square root of the ratio of the elastic modulus *E* and surface energy γ to the crack length *a*, expressed as

(18) $$\begin{array}{c} \sigma_{c}\propto \sqrt{\frac{E\gamma }{a}} \end{array}$$

where *a* is the half-length of the crack for a central crack in an infinite plate. The proportionality constant in Griffith’s criterion depends on the geometry of the deformed specimen and the morphology of the crack. Although this constant varies based on specific conditions, it generally remains in the order of unity. This analogy between Griffith’s criterion and the phase-field formulation reinforces the physical consistency of the phase-field modeling of fracture, as the parameter *A* must have the same γ/ℓ-dependence and magnitude as *B* for the phase-field criterion to remain consistent with Griffith’s theory (recall Equation (17)). Hence, for the phase-field fracture criterion to align with Griffith’s, the free model parameter *A* should have the same magnitude as *B*. Subsequently, the parameter *A* value is chosen here as 1.5×B. However, the current model criterion given by Equation (16) is generic and could possibly describe non-ideal brittle or quasi-brittle fracture, where Griffith’s criterion usually fails. Equation (17) reveals an inverse square-root dependence on the length scale, which indicates that the phase-field critical stress inherits the same scaling behavior as Griffith’s criterion, following an inverse square-root dependence on crack length [26]:

(19) $$\begin{array}{c} \sigma_{c}\propto \frac{1}{\sqrt{a}}\;\left(\mathrm{Griffith}\right) \end{array}$$

Or, equivalently,

(20) $$\begin{array}{c} \sigma_{c}\propto \frac{1}{\sqrt{\ell }}\;\left(\mathrm{Phase}-\mathrm{field}\right) \end{array}$$

Recent experimental micro-mechanical tests have confirmed this size-dependence for UO_2_, validating theoretical predictions [27].

#### 2.1.2. Mechanical Equilibrium Equations

In this study, the deformation of uranium dioxide (UO_2_), a brittle material, is modeled using the infinitesimal strain theory framework. This approach is well suited for brittle materials like UO_2_, as they generally undergo small, elastic strains before failure. The framework effectively captures the mechanical behavior of solid bodies under such small deformations. Consequently, the material response is assumed to be linear, with elastic strain being the only strain considered. The mechanical equilibrium equation is given by

∇·σ=0

where σ is the Cauchy stress tensor. For a brittle material governed by Hooke’s law,

(21) $$\begin{array}{c} \sigma =C\epsilon_{elastic} \end{array}$$

where C is the fourth-order elasticity tensor and $\epsilon_{elastic}$ is the elastic strain. For isotropic materials, C depends solely on two material properties: the elastic modulus (*E*) and Poisson’s ratio (ν). To simplify, the stress–strain relationship is often expressed directly in terms of *E* [28]. Equation (21) reduces to

(22) $$\begin{array}{c} \sigma =E\epsilon_{elastic} \end{array}$$

$\epsilon_{thermal}$ denotes the thermal expansion strain and ϵ is the localized strain obtained from the gradients of the displacement vector:

(23) $$\begin{array}{c} \epsilon =\epsilon_{elastic}+\epsilon_{thermal} \end{array}$$

(24) $$\begin{array}{c} \epsilon =\frac{1}{2}\left[\nabla u+{\left(\nabla u\right)}^{T}\right] \end{array}$$

Here, u represents the displacement vector, ∇ is the gradient operator, and ${\left(\cdot \right)}^{T}$ indicates the transpose of a tensor. The elastic modulus *E* is defined as

(25) $$E=I\left(\eta \right)E^{0}+\left(1-I\left(\eta \right)\right)E^{1}$$

where $E^{0}$ and $E^{1}$ correspond to the elastic modulus of the damage-free and fully damaged states, respectively. A very small elastic modulus is assigned to the fully damaged state to effectively represent negligible stiffness. I(η) interpolates the stiffness between the damaged and undamaged states, e.g., the intact solid and crack phases. This interpolation function, often called the degradation function, is represented by I(η) in this work, making the strain energy a function of the phase-field variable η.

#### 2.1.3. Heat Equation and Thermal Conductivity Correlation

UO_2_ fuels expand based on their thermal expansion coefficient, creating stress-free strain (eigenstrain). Stress arises only when there is constraint or exposure to uneven temperature gradients. Therefore, the phase-field fracture model is coupled with the heat conduction equation to enable the simulation of fractures induced by thermo-mechanical loading. The heat conduction equation is essential for determining the temperature gradient within the fuel pellet. It is represented as

(26) $$\begin{array}{c} \rho C_{p}\frac{dT}{dt}=k\nabla^{2}T+Q \end{array}$$

Here, *k* denotes thermal conductivity, *T* represents temperature, ρ and $C_{p}$ correspond to density and specific heat, respectively, while *Q* signifies the heat source. For pellet-scale simulations, a Dirichlet boundary condition is imposed on the outer edge of the pellet to represent the coolant temperature. Furthermore, a zero-flux boundary condition is applied at the pellet’s centerline to ensure a comprehensive and physically consistent solution. The Wiesenack model [29,30] represents the burnup effect on the thermal conductivity of uranium dioxide pellets as the result of fission product accumulation within the UO_2_ matrix. These fission products disrupt thermal conduction in the fuel lattice by increasing phonon–phonon collisions, thereby reducing thermal conductivity. In this context, the heat source is defined by the target rod power level, primarily affecting the centerline temperature. Thermal conductivity in this work is temperature- and burnup-dependent and can be expressed by the following relationship:

(27) $$\begin{array}{c} k\left(T\right)=\frac{1}{\left(A_{0}+A_{1}\cdot BU\right)+\left(B_{0}+B_{1}\cdot BU\right)\cdot T}+C\cdot \exp\left(D\cdot T\right) \end{array}$$

where *BU* is fuel burnup (GWd/tU). The parameter values are provided in the Table 1.

Eigenstrain refers to a strain field introduced into the model that does not directly result from external mechanical loads, e.g., stress-free strain. In this work, eigenstrain is specifically used to account for thermal expansion strain caused by temperature changes. As the pellet is heated or cooled, eigenstrain causes expansion or contraction, inducing stresses when thermal gradients are present. Eigenstrain is calculated using the following formula for thermal expansion strain:

(28) $$\begin{array}{c} \epsilon_{\mathrm{thermal}}=\alpha \left(T-T_{\mathrm{ref}}\right) \end{array}$$

where α is the coefficient of thermal expansion, *T* is the current temperature (in Kelvin), and $T_{\mathrm{ref}}$ is the reference temperature at which the material is free of thermal strain. (298 K in this work). By incorporating Equation (28) into Equation (23), the effects of thermal expansion are integrated into the strain experienced by the system, which, in turn, influences the calculation of stress within the phase-field formulation. The resulting partial differential equations (PDEs) are solved using the finite element method (FEM) with implicit time integration and the Preconditioned Jacobian-Free Newton–Krylov (PJFNK) nonlinear solver, implemented within the open-source Multiphysics Object-Oriented Simulation Environment (MOOSE) [31].

#### 2.1.4. Model Parameters

The material properties used in this study are summarized in Table 2, which includes key parameters critical for modeling the behavior of UO_2_ fuel under transient conditions. The fracture stress ($\sigma_{f}$) is taken as 200 MPa, and the elastic modulus (*E*) is set to 200 GPa, both derived from experimental studies [27]. Poisson’s ratio (ν) is specified as 0.33 [32]. Thermal conductivity (*k*) follows the Halden correlation as defined in Equation (27) [29]. Additionally, the density (ρ) is 10,970 ${\mathrm{kg}/m}^{3}$ [33], and the specific heat capacity ($C_{p}$) is 480 J/kg·K [34]. The thermal expansion coefficient (α) is $10^{-5}\;K^{-1}$ [21]. All these properties were derived from experimental data to ensure the realistic and accurate simulation of material behavior. The model parameters in Table 3 were calculated using material properties and the equations defined in the Section 2.1.1.

## 3. Results and Discussion

### 3.1. Benchmark Studies of Crack Initiation and Propagation

In this section, we describe how benchmark studies were conducted to evaluate crack initiation and propagation processes. For simplicity, 2D square domains were chosen for these sets of simulations. Tension was applied to the top boundary, while the bottom boundary was constrained to zero displacement, effectively simulating tensile loading. These sets of simulations also demonstrated the capabilities of the novel stochastic phase-field fracture approach presented here (recall Equation (2)). Particularly, we showed that the model was able to separately simulate crack nucleation and propagation processes and predict distinct threshold values of stress for each. Moreover, we also showed that the stochastic term in the evolution equation (Equation (2)) was sufficient to capture the stochastic nature of fracture and the resultant variations of fracture patterns.

**Figure 1:**
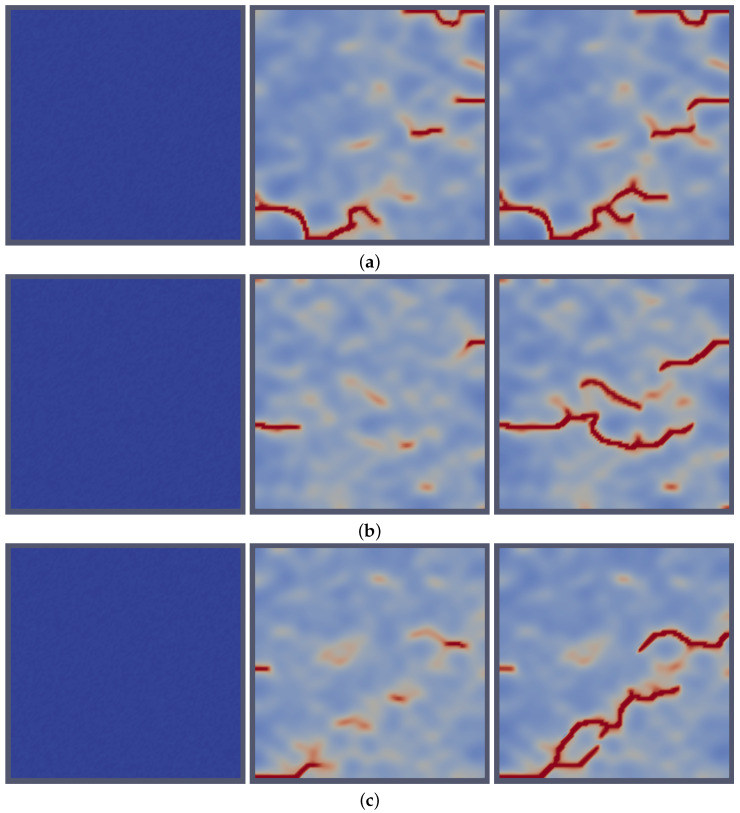
Realizations of the distinct initiation and propagation of cracks in initially crack-free domains using the stochastic phase-field fracture approach implemented here. The figure illustrates crack development over increasing time steps for three different initial-condition cases, (**a**–**c**), under the same applied stress. For each initial condition, time steps progress from left to right.

Figure 1 displays crack initiation in a defect-free domain, demonstrating the model’s ability to simulate the nucleation of cracks without the need for pre-existing defects/cracks. This capability highlights the advantage of the approach introduced here that treats fracture as a stochastic phase transition. Furthermore, this treatment enables the direct accounting of the stochastic effects on the fracture process. This is evident from Figure 1a–c, showing distinct fracture patterns with different cracks’ paths for the same applied value of stress. This is possible via either choosing different random distributions of the stochastic term or simply in the initial random, small values of the phase-field variable. The latter method was employed in those simulations with a uniform random distribution of η between +/−0.01, representing a solid with underlying defects such as porosity or dislocations but not cracks. It is worth noting that the stochastic nature of fracture and the distinct patterns of cracks were observed in UO_2_ pellets [35,36,37,38,39].

To determine the stress threshold required to sustain crack growth, a series of incremental tests were conducted and are summarized in Table 4. In this analysis, the applied stress was systematically decreased in successive iterations to identify the minimum stress at which crack propagation occurs.

**Figure 2:**
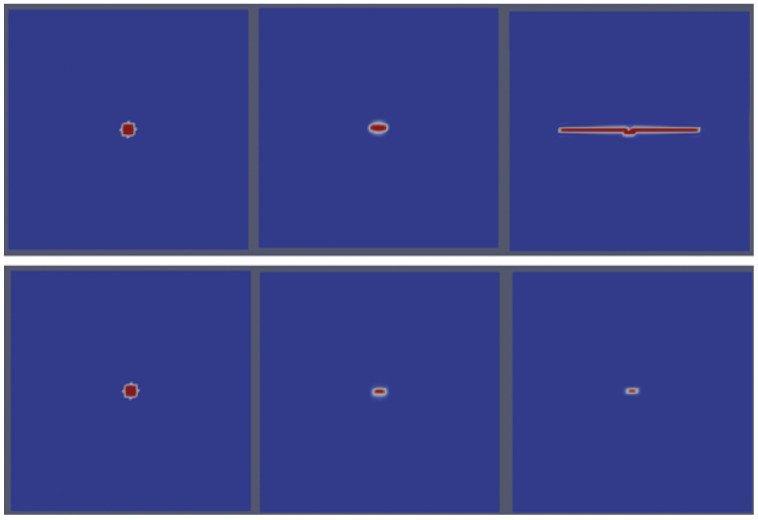
An illustration of crack propagation in the pre-existing-crack case. (**Top row**): a demonstration of crack growth under sufficient strain energy, where cracks propagate horizontally, normal to the applied tension. (**Bottom row**): a depiction of the absence of crack propagation when the strain energy is insufficient, leading to crack collapse and disappearance.

The data reveal that crack propagation occurs at an applied stress as low as 67 MPa, with a corresponding strain of 0.000335 and strain energy of 11,222.5 Pa. Below this stress threshold, crack growth is not sustained. This critical value is approximately one-third of the intrinsic (crack-free) fracture stress used in this study, highlighting the stress sensitivity of the crack propagation process. The results provide quantitative insight into the relationship between applied stress, strain energy, and crack behavior. Figure 2 illustrates qualitatively two distinct cases of the pre-existing crack benchmark. The top row of Figure 2 visually depicts the propagation of a pre-existing crack under sufficient strain energy conditions. In this scenario, the crack propagates horizontally, perpendicular to the direction of the applied tension. This propagation is driven by the availability of sufficient energy to overcome the resistance of the material, enabling crack growth along the planes of maximum stress concentration. In contrast, the bottom row of Figure 2 demonstrates a case where the strain energy is insufficient for sustained crack propagation. As a result, the crack fails to grow and eventually collapses or disappears. This outcome highlights the critical role of strain energy in determining the stability and propagation of cracks within the UO_2_ fuel pellet.

We now turn our attention to fracture in UO_2_ pellets. Beyond this point, pellet-scale simulations were investigated to provide relevant analysis. The mesh employed was a two-dimensional circular domain with a radius of 4 mm. Based on the one-third ratio, defined as h=l/3, the length scale (*l*) was set to 0.3 mm, resulting in an element size (*h*) of 0.1 mm.

**Figure 3:**
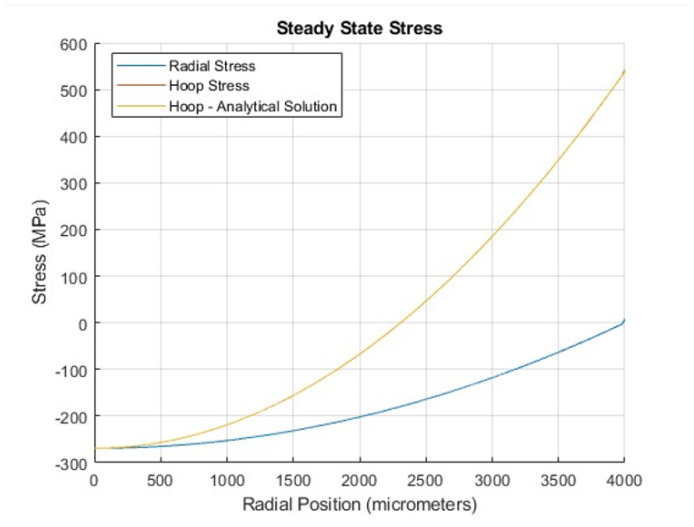
Hoop (tangential) and radial stress profiles as functions of radial position in steady-state stress. The figure demonstrates that the analytical solution for the hoop stress aligns precisely with the numerical results.

To validate the initial calculations, analytical solutions for temperature and hoop stress were used for comparison. Assuming temperature-independent thermal conductivity, the radial temperature profile in the fuel is expressed analytically as follows [38]:

(29) $$\begin{array}{c} T\left(r\right)-T_{S}=\frac{QR^{2}}{4k}\left(1-\frac{r^{2}}{R^{2}}\right) \end{array}$$

where $T_{S}$ is the fuel surface temperature, *T* is the temperature, *r* is the radial position in the pellet, *k* is the fuel thermal conductivity (W/m·K), and *Q* is the volumetric heat generation rate (W/m^3^). The hoop stress profile in the fuel is expressed as follows [38]:

(30) $$\begin{array}{c} \sigma_{\theta }\left(r\right)=\frac{-\alpha EQR^{2}}{16(1-\nu^{2})k}\left(1-\frac{3r^{2}}{R^{2}}\right) \end{array}$$

where α is the coefficient of thermal expansion of UO_2_, *E* is the elastic constant of UO_2_, and ν is its Poisson ratio. Figure 3 presents the steady-state hoop and radial stress profiles as a function of radial position. The average hoop stress was determined to be 134.63 MPa, while the average analytical hoop stress was calculated as 134.60 MPa. The relative error between the numerical and analytical results is 5.33 ×10^−9^, confirming excellent agreement.

**Figure 4:**
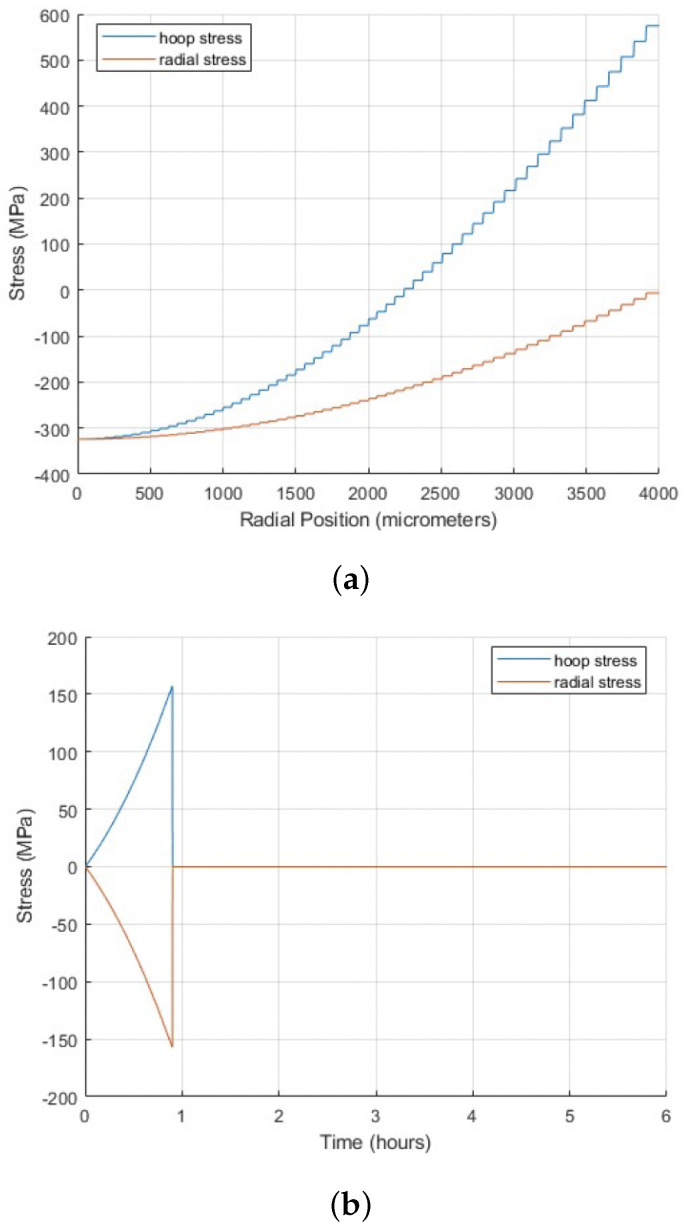
(**a**) Average hoop (tangential) and radial stress profiles as functions of radial position during the transient simulation, captured in the time step just before fracture occurred (0.9 h), highlighting the conditions leading to crack nucleation. (**b**) Average hoop (tangential) and radial stress profiles as functions of time during the transient simulation, highlighting the significant decline at 0.9 h associated with crack nucleation initiation.

Figure 4 shows the results of an analysis that examined the stress distribution within the pellet just before fracture occurred (at 0.9 h) during a transient simulation. Figure 4a shows average hoop (tangential) and radial stress profiles as functions of radial position during the transient simulation, captured in the time step just before fracture occurred (0.9 h). Radial stress stayed compressive throughout the entire domain, whereas hoop stress was compressive at the center and became tensile toward the edge. Figure 4b shows that, before fracture, the tangential and radial stresses exhibited equal magnitudes but opposite signs, indicating balanced stress behavior where the forces in different directions counteracted each other, maintaining equilibrium within the pellet.

**Figure 5:**
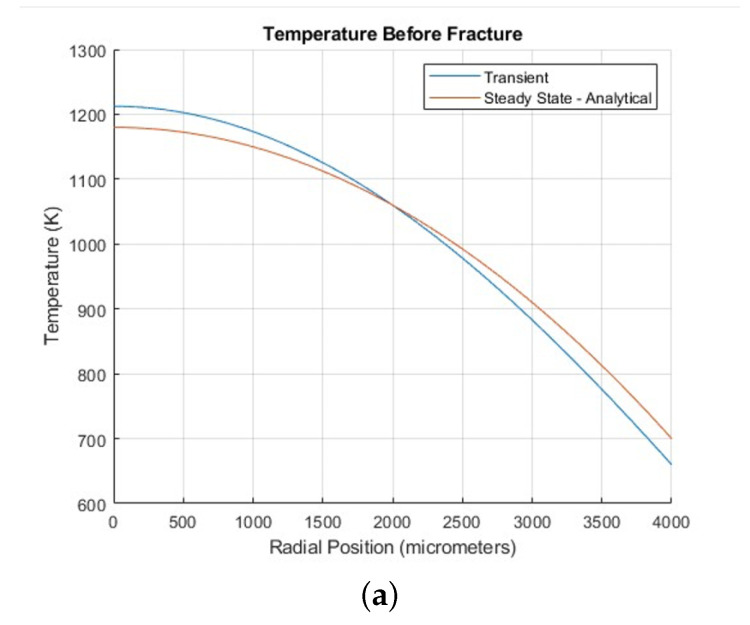

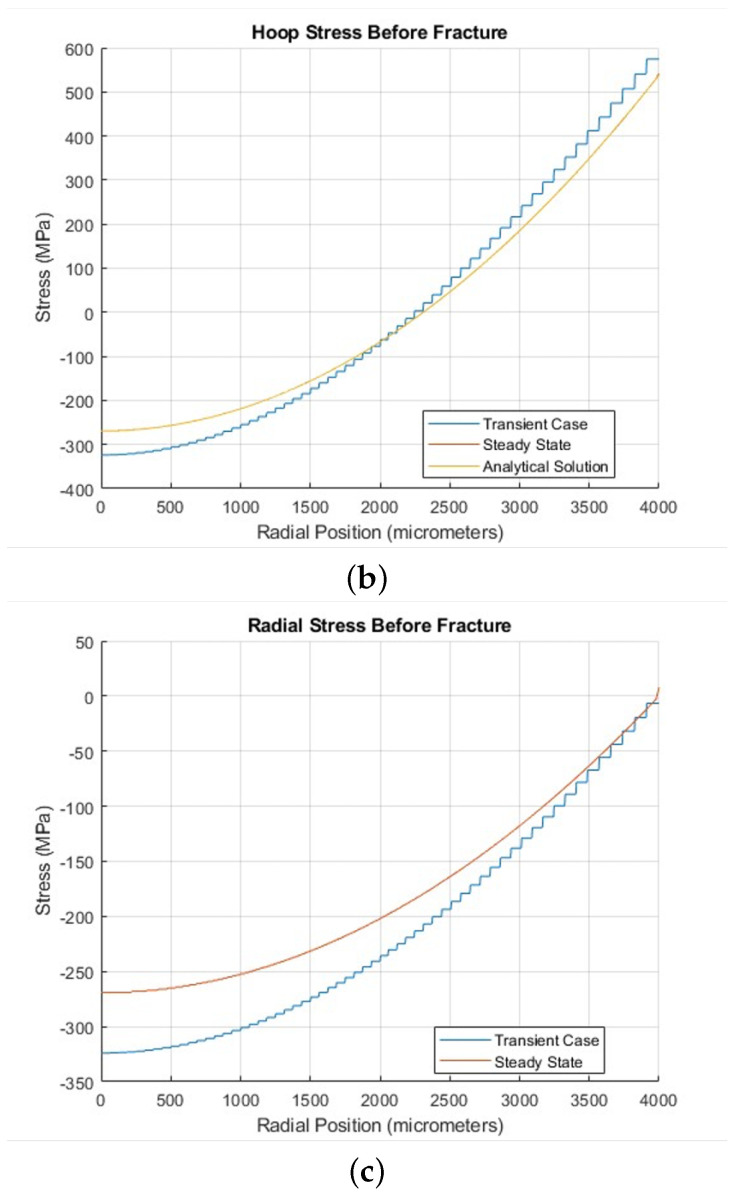
Comparison of transient and steady-state cases: (**a**) Steady-state temperature as a function of radial position compared to the start-up transient (detailed in Section 3.2). (**b**) Average hoop (tangential) stress profiles as functions of radial position during the transient simulation. (**c**) Radial stress profiles as functions of radial position during the transient simulation. All figures were captured in the time step just before fracture occurred (0.9 h).

We also discuss here the major differences between the steady and transient solutions for the case of a constant thermal conductivity to elucidate the basic characteristics of the transient solutions where analytical expressions cannot be obtained. A detailed description of the transient simulations with variable thermal conductivity and burnup effects will be discussed later. Figure 5 displays the spatial distributions of hoop and radial stresses by comparing the start-up transient profiles (see Section 3.2 below) at 0.9 h with steady-state benchmark results. Figure 5a depicts that the stress distributions align closely with the steady-state predictions, demonstrating the model’s reliability. However, the slightly higher temperature gradient at 0.9 h results in larger stress magnitudes and reflects the influence of transient thermal conditions. Figure 5b,c show hoop and radial stress distributions as functions of radial position during both transient and steady-state simulations. Similarly, slight differences are observed at 0.9 h, once again highlighting the impact of transient thermal conditions.

### 3.2. Transient Conditions: Start-Up and Power Ramp Phase

**Figure 6:**
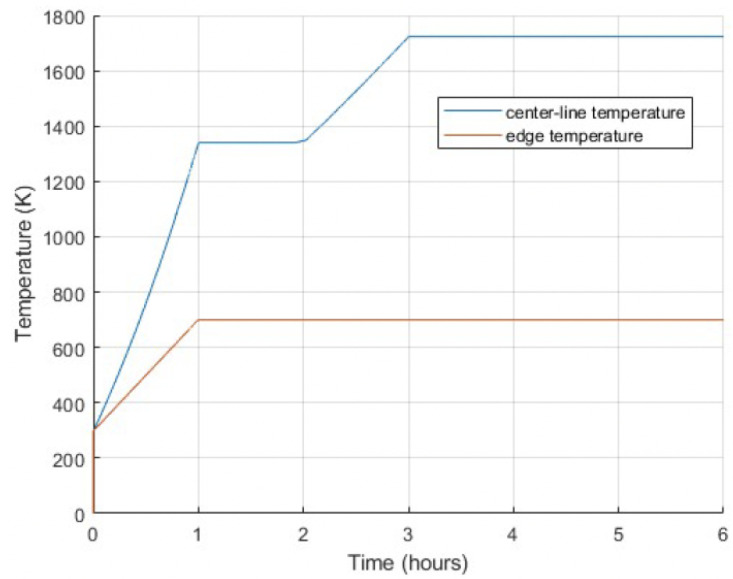
Temperature as a function of time during the start-up and power ramp phases, showing centerline and edge temperatures over the 6 h simulation period.

The model incorporates a transient composed of two distinct phases: the start-up phase and the power ramp phase. Figure 6 presents the timeline of events, with centerline and edge temperatures plotted over time. Slanted lines represent phase transitions, while horizontal lines indicate holding periods during steady-state conditions. The start-up phase was designed to be consistent across all tests. It featured a constant heat rate, increasing the centerline temperature from 298 K to 1340 K over the first hour, followed by a 1 h hold period. By the end of this phase, the centerline temperature stabilized at approximately 1340 K, while the edge temperature remained below 700 K, reflecting effective boundary heat dissipation. The power ramp phase began after 2 h and spanned 4 h. After the 4 h duration of the power ramp phase, the final crack pattern was established. It captured the fracture behavior under transient thermal conditions. This setup effectively modeled transient conditions. For clarity, Figure 6 illustrates a single case scenario where the temperature reached 1730 K, providing a clear depiction of the start-up and power ramp phases. In this work, based on this transient condition setup, the system will be analyzed considering different temperatures and heating rates in the following sections. The transient conditions will also be discussed in detail.

**Figure 7:**
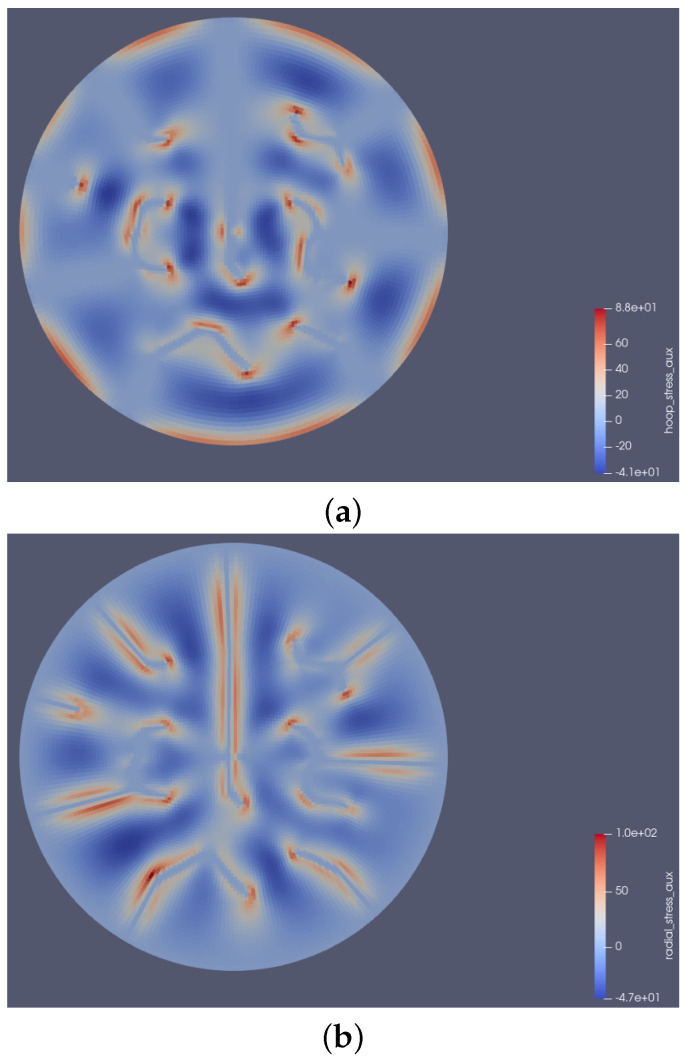
Final stress profiles at the end of the power ramp: (**a**) Hoop (tangential) stress. (**b**) Radial stress.

In Figure 7, the hot contours (regions with warmer colors like red) represent areas of higher stress magnitudes in both hoop (tangential) and radial stress profiles. The hot zones in both cases indicate critical stress locations where crack growth or propagation was most likely to occur. At the end of the power ramp, it was observed that at the crack tipped, hoop stress dominated (Figure 7a), while along the crack, radial stress took precedence (Figure 7b). The dominance of hoop stress at the crack tips determined the crack direction. Understanding this stress distribution is essential for accurately predicting crack patterns because most models in the literature emphasize radial cracks while neglecting circumferential cracks that may develop during operational power ramps [39]. The irregular distribution of peak hoop stress highlights the non-uniform nature of stress across the domain in Figure 7a. This suggests a network crack pattern, characterized by interconnected cracks forming a complex and irregular distribution across the radial position.

### 3.3. Modeling Distinct Fracture Patterns in UO_2_ Pellets

Various cracking patterns have been reported for both fresh and irradiated UO_2_ pellets [21,22,35,36,37,38,39]. These have been attributed to the different microstructures of those pellets, distinct operating/transient conditions, or irradiation effects. Four distinct strategies were employed here to appropriately account for those effects: first, the utilization of a time-dependent stochastic term (recall Equation (2)) to capture the evolution of microstructural features; second, accounting for the initial variable microstructural characteristics of the fuel by using random initial conditions for the phase-field variable; third, evaluating the effect of burnup on thermal conduction and consequently thermal fracture patterns; and lastly, considering the influence of the main thermal transient conditions such as the heating rate and final temperature of the pellets.

#### 3.3.1. Incorporating the Effect of Randomness in Microstructure Evolution

Previous experimental studies have demonstrated that the strength of UO_2_ is highly influenced by its porous microstructure. [40] demonstrates that defect mobility and recovery mechanisms vary with temperature, with significant recovery stages observed between 773 and 1273 K and above 1273–1373 K. These studies emphasize that the thermal recovery of extended defects in polycrystalline UO_2_ is a complex process governed by temperature and irradiation conditions. They also reveal that microstructural characteristics such as the size, shape, and distribution of pores play a significant role in UO_2_ fracture under thermo-mechanical and irradiation conditions. While advanced modeling and characterization techniques could predict or measure the average density and size of these features, precise descriptions of their spatial and size distributions are much more complex to quantify. In our model formulation, we can account for these uncertainties directly using the stochastic term that appears in the evolution equation (Equation (2)).

**Figure 8:**
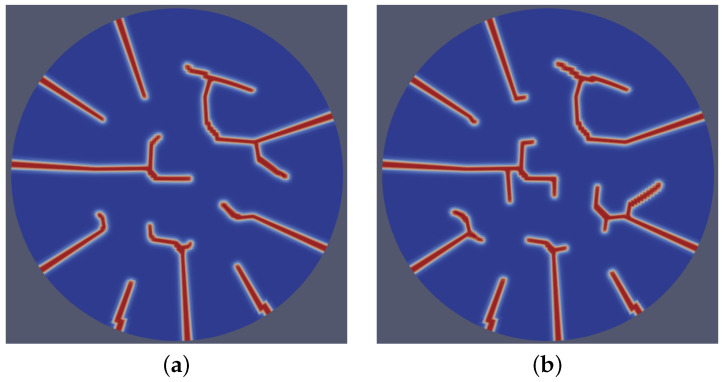
Fracture patterns resulting from two distinct cases (**a**,**b**) of dynamic stochastic distributions of underlying microstructural evolution, highlighting variations in crack morphology and propagation pathways due to differing stochastic conditions.

A dynamic stochastic field was utilized to represent the inherent randomness in microstructural features during the time-dependent microstructure evolution processes. To implement this numerically, we initialized λ (see Equation (2)) for each time step using a random number stream with a seed derived as a function of the time step number at the start of every residual calculation. In each realization, the value of λ was drawn from a uniform random distribution in the range of −0.01 to 0.01 of the the average strain energy density. This approach facilitated the accurate modeling of probabilistic effects while ensuring numerical stability. By performing so, we introduced a degree of randomness to the model, enabling it to account for variations due to probabilistic changes in the microstructure. The inclusion of variable stochastic terms in the simulations introduced distinct variations in the fracture patterns. As shown in Figure 8, the distinct stochastic cases demonstrated varying degrees of irregularity and branching in the fracture paths, highlighting the sensitivity of the model to the stochastic terms. Figure 8a displays relatively smoother crack propagation and a more deterministic fracture behavior. In contrast, Figure 8b exhibits more pronounced stochastic effects, leading to increased complexity in the crack morphology, characterized by a higher frequency of branching and deviation from linear paths. The model effectively demonstrated the qualitative impacts of microstructural characteristics.

#### 3.3.2. Accounting for the Initial Microstructural Characteristics

**Figure 9:**
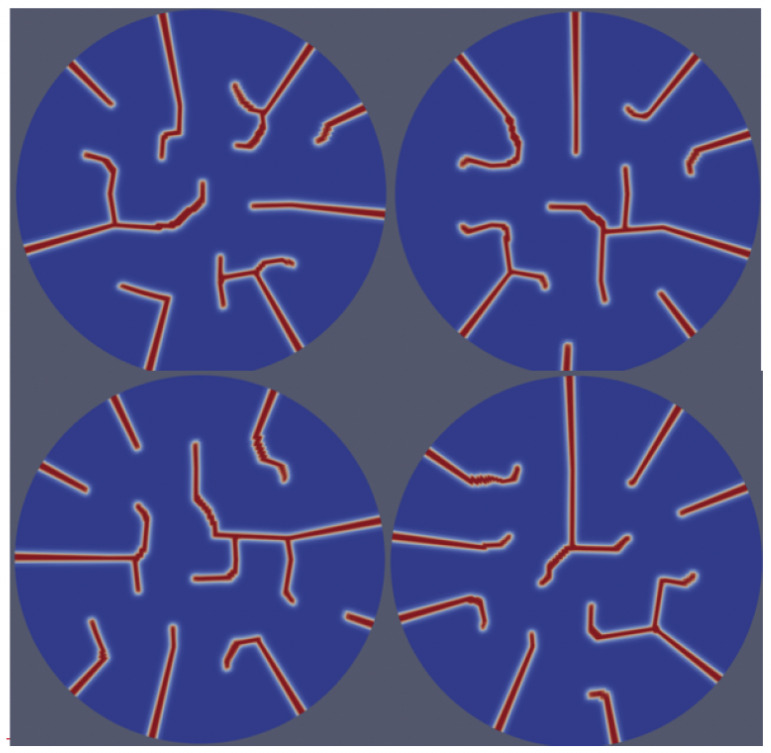
Fracture patterns under varying initial conditions in η, representing distinct initial microstructures, illustrating their influence on crack initiation and propagation behavior and resulting in four distinct fracture morphologies.

Another source of variability and randomness in the microstructure of fuel pellets originates from manufacturing processes [41]. As discussed above (recall Section 3.1), this can be accounted for by initiating the η variable of the intact solid phase with small non-zero values, representing low densities of underlying defects/microstructural features with variable distributions. In the simulations presented here, values of η ranging between 0.0005 and 0.01 were used. Figure 9 shows the influence of varying initial conditions on fracture behavior, revealing significant differences in crack propagation and morphology. Comparing four different initial configurations, crack initiation points varied across cases, all exhibiting edge-dominated initiation. Propagation paths were distinctly affected, with certain simulations producing highly branched and irregular trajectories, while others exhibited more linear and organized patterns. Notable differences in branching behavior were observed, with some cases generating complex networks featuring numerous branches. Fracture symmetry also varied, with patterns ranging from highly symmetrical to markedly asymmetrical, reflecting the model’s sensitivity to initial conditions. The variations can be explained by the choice of initial conditions. Quantitatively, disparities in total crack length, branching density, and fractured area further highlight the model’s capability to capture the significant influence of initial conditions, representing variant initial microstructures, on fracture development. These results are valuable because experimental work by Radford [41] reported the influence of fabrication parameters and microstructural characteristics on the mechanical strength of UO_2_ fuel pellets. This, in turn, has a profound impact on their fracture behavior. Critical to note is that, as shown in Figure 9, the number of cracks varied with the initial condition, accurately reflecting physical behavior and aligning with experimental data. This marks a key advancement over prior models constrained by fixed-number crack strategies and demonstrates the model’s effectiveness in implicitly accounting for the underlying microstructures and their variations.

#### 3.3.3. Effect of Burnup on Fracture Patterns

**Figure 10:**
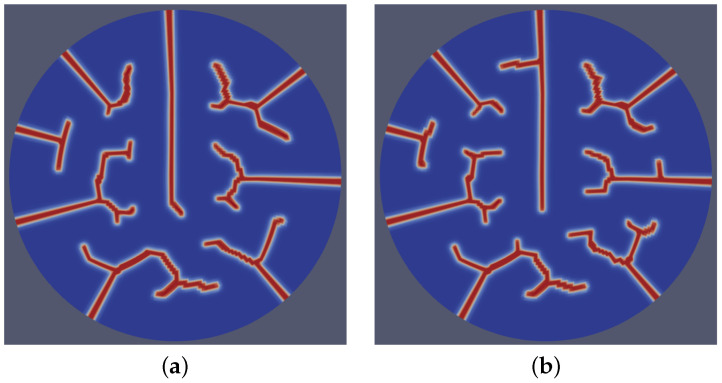
Comparison of fracture patterns under different heat rates: (**a**) <1 K/s exhibits smoother and less intricate crack propagation and (**b**) 100 K/s shows more complex and irregular branching.

Increasing burnup in fuels is known to strongly affect their thermo-mechanical properties and their fracture behavior [34,35,36,37,38,39,40,42]. To investigate this scenario here, we conducted simulations of high-burnup fuels and compared them to reference cases of fresh fuels. The local burnup was increased to 80 GWd/tU at the onset of the power ramp phase (recall Figure 6). This effect led to a reduction in thermal conductivity (recall Equation (27)) and a corresponding rise in the centerline temperature, with the final centerline temperature exceeding 2600 K. Figure 10a corresponds to a heat rate of <1 K/s, while Figure 10b represents the case with a heat rate of 100 K/s. The comparison of the two cases highlights distinct differences in fracture propagation and morphology. Figure 10b exhibits a higher degree of irregularity in crack patterns, with more frequent branching and nonlinear pathways. Compared to fresh fuels (Figure 9), the fracture patterns of high-burnup fuels display a larger number of cracks, more complex geometries in cracks, and more pronounced branching and propagation in non-radial directions.

**Figure 11:**
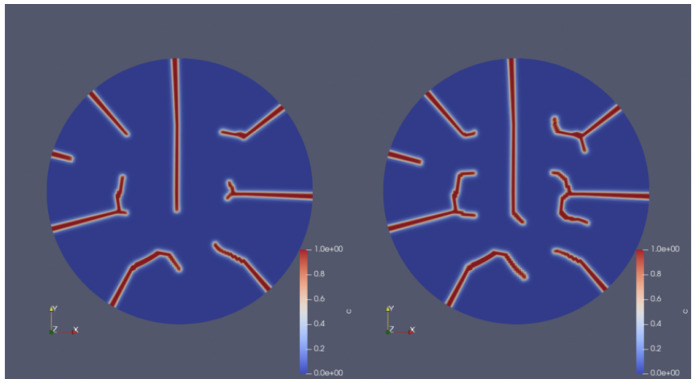
Crack profiles of a UO_2_ fuel pellet illustrating the propagation of a crack under varying operational phases. (**Left**): the crack profile of the UO_2_ fuel pellet after the start-up phase at t=1.99 h. (**Right**): the crack profile of the same fuel pellet after the power ramp phase at *t* = 6 h.

Figure 11 displays the evolution of crack patterns over time, transitioning from isolated radial cracks to a complex interconnected network under varying operational phases. The left image (at t = 1.99 h) shows an early-stage crack network with fewer isolated cracks. The cracks are primarily linear and localized, suggesting that the stress conditions initiated cracking but had not yet fully propagated. The right image (at t = 6 h) displays a more developed crack network with an increased number of cracks and greater connectivity. The cracks are longer and branching is more prominent, indicating advanced propagation during the power ramp phase. The transition from isolated cracks to a more extensive network suggests that the fuel integrity was progressively compromised during the power ramp phase. In the right image, when the first crack reaches the center and propagates to one side, the crack on that side appears to retract or stop propagating further. This behavior might be the result of stress relief in that region, as the crack reaching the center alleviates the localized tensile stress. Consequently, the crack on that side may lose the driving force required for further propagation in that direction. Additionally, when multiple cracks are present, their stress fields can overlap, leading to a phenomenon known as stress shielding. This interaction reduces the stress intensity at the tips of some cracks, preventing them from extending further toward the center.

#### 3.3.4. Crack Propagation Patterns in a UO_2_ Fuel Pellet at Different Heating Rates and Temperatures

We now turn our attention to the influence of the severity of the transients on the fracture morphologies. Here, the parameters that describe the severity of the transients are the heating rates and pellet temperatures. To model the in-reactor fracture behavior of UO_2_, temperature transients with different profiles were used to simulate a wide range of reactor conditions. The resulting fracture patterns were then analyzed under these varying transients. Each transient consisted of two distinct stages: a start-up phase and a power ramp phase. The start-up phase was standardized across all transients, spanning a total duration of two hours. During the first hour, the centerline temperature increased linearly from 298 K to 1340 K at a rate of approximately 0.2 K/s, while the outer edge temperature concurrently rose from 298 K to 700 K in a linear progression. The edge temperature was consistently maintained below 700 K throughout this study.

The temperature distribution across the domain adhered to a parabolic profile, derived from the heat conduction equation. Following the initial hour of temperature ramping, a 1 h holding period was implemented. During this holding period, the centerline temperature was stabilized at 1340 K, and the outer edge temperature remained fixed at 700 K. This steady-state phase facilitated the nucleation and propagation of cracks to their maximum extent, marking the completion of the start-up phase.

The start-up phase was followed by the power ramp phase, designed to investigate the fuel’s behavior under varying transient conditions. This phase began with a linear escalation in fuel power, governed by a time-dependent heat source, resulting in a proportional linear increase in the centerline temperature of the fuel. The rate of this temperature increase represented the first variable of interest, with heat rates ranging from below 1 K/s to 100 K/s. Despite variations in the centerline heat rate, the edge temperature consistently remained fixed at 700 K.

The power ramp phase continued until the centerline temperature reached a predetermined target, which constituted the second variable in this study. Three specific target temperatures—1730 K, 2006 K, and 2302 K—were selected based on a range suitable for comparative analysis. This phase extended until the cumulative simulation time reached 6 h. Over this period, cracks propagated until the available free energy was depleted, at which point their growth ceased. By systematically combining these variables, a total of 15 transients were examined, generating a robust dataset for comprehensive analysis.

**Figure 12:**
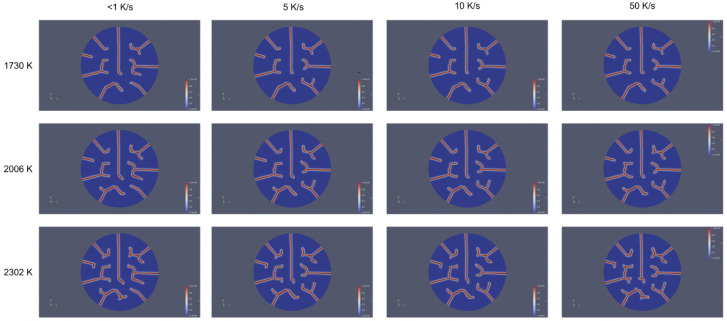
Crack propagation patterns in a UO_2_ fuel pellet at different heating rates (<1 K/s, 5 K/s, 10 K/s, and 50 K/s) and temperatures (1730 K, 2006 K, and 2302 K). The figure highlights the combined effects of heating rate and temperature on crack morphology within the microstructure.

Figure 12 shows crack propagation patterns in a UO_2_ fuel pellet at different heating rates (<1 K/s, 5 K/s, 10 K/s, and 50 K/s) and temperatures (1730 K, 2006 K, and 2302 K). Cracks consistently initiated at the edge of the pellet and propagated radially inward toward the center in all cases. This pattern was primarily driven by the thermal gradient. The influence of heating rate was evident as, at low heating rates (<1 K/s), cracks propagated radially inward in a straight, linear pattern toward the center. At high heating rates (50 K/s), cracks still propagated inward from the edge to the center but often exhibited branching or deviation near the center. Higher heating rates generated steeper thermal gradients, resulting in greater localized stress at crack tips. Consequently, some cracks branched near the center, deflected, or interacted with stress fields from neighboring cracks, leading to a more complex propagation pattern. At lower heating rates, the thermal gradients were less steep, and stress fields were more evenly distributed. The effect of heating rate led to more aggressive crack propagation, as evidenced by increased crack density, length, and branching at 50 K/s compared to <1 K/s for any given temperature. The influence of temperature was also significant. At lower temperatures (1730 K), cracks propagated radially inward in a direct trajectory due to reduced strain energy and stress distribution. However, at higher temperatures (2302 K), elevated temperatures increased thermal expansion and strain energy, causing stress redistribution. This redistribution led to additional crack growth along circumferential directions and potential interactions with other cracks near the center. These trends in the fracture patterns compare favorably to available out-of-pile experimental data [21,43,44].

**Figure 13:**
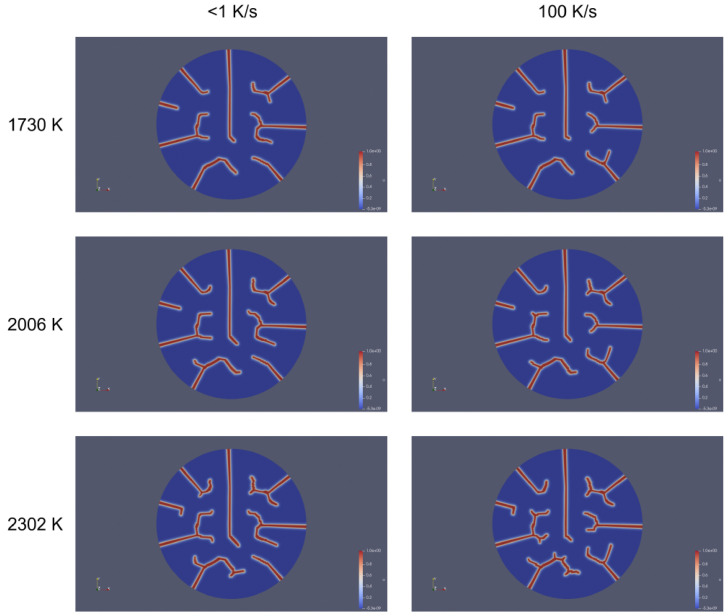
A comparison of crack propagation patterns in a UO_2_ fuel pellet at two distinct heating rates (<1 K/s and 100 K/s) and temperatures (1730 K, 2006 K, and 2302 K). The figure emphasizes the contrasting crack morphologies observed at the lowest and highest heating rates.

In general, the heating rate dictated the steepness of the thermal gradient, influencing crack branching and deflection near the center. Temperature provided strain energy for longer cracks and promoted circumferential propagation or interaction with other cracks, particularly at high temperatures combined with high heating rates. Figure 13 shows a comparison of crack propagation patterns in a UO_2_ fuel pellet at two distinct heating rates (<1 K/s and 100 K/s) and temperatures (1730 K, 2006 K, and 2302 K). While temperature increased the overall strain energy and crack-driving forces, the heating rate had a more direct and localized impact on crack morphology by intensifying thermal gradients and stress concentrations. This dominance is evident in Figure 13, where at lower heating rates (<1 K/s), cracks were sparse and linear even at higher temperatures (2302 K). In contrast, at high heating rates (100 K/s), cracks became highly branched and interconnected, even at moderate temperatures (2006 K).

## 4. Concluding Remarks

This study introduced an advanced thermo-mechanical phase-field fracture model to explore the fracture behavior of high-burnup uranium dioxide (UO_2_) fuel under transient reactor conditions. An innovative aspect of the model is the treatment of fracture as a stochastic phase transition via the explicit incorporation of a stochastic term in the kinetic evolution equation of cracks. This directly accounts for the underlying microstructural heterogeneities, which in turn results in variable fracture strength and distinct crack patterns. The model also takes into consideration the effects of operational and transient conditions along with the degradation of thermal conductivity with temperature and burnup.

The findings reveal that high burnup significantly influences the fracture behavior of UO_2_ fuel, resulting in increased crack density, pronounced branching, and more irregular trajectories compared to fresh fuels. Furthermore, transient conditions, such as heating rates and temperatures, play a pivotal role in determining the morphology of cracks. Specifically, steep thermal gradients at higher heating rates are shown to amplify localized stress concentrations, leading to complex and interconnected crack networks. The reduction in thermal conductivity observed in high-burnup fuels intensifies these temperature gradients, thereby exacerbating both radial and circumferential cracking, which poses challenges to fuel integrity.

The model’s predictive accuracy was validated against analytical benchmarks and experimental data, affirming its reliability for studying fracture behavior in irradiated nuclear fuels. By incorporating stochasticity to capture microstructural heterogeneities and transient operational conditions, this work provides a robust framework for evaluating the mechanical performance and safety of nuclear fuels. The insights gained are particularly relevant for assessing the implications of extending burnup limits in nuclear reactors, offering critical contributions to fuel design and safety analyses. This study underscores the potential of advanced modeling techniques in supporting nuclear reactor safety evaluations and guiding the design of next-generation fuel systems.

Several future directions can be pursued to extend the model’s capabilities and improve its quantitative predictions. First, the magnitude of the stochastic term representing the heterogeneity of the microstructure and/or irradiation effects could be derived on a more physical basis. For instance, its value could be linked to the strain energy associated with concentrations of point and extended defects or microstructural features. Second, thermal and irradiation creep relationships should be incorporated to accurately account for stress relief from these mechanisms, which would ultimately reduce the accumulated thermal stresses available to induce fracture. Third, the exact radial dependence of burnup and the resulting heterogeneous degradation of thermal conductivity should be fully considered for a more realistic description of the state of high-burnup fuels. Fourth, while our transient simulations primarily focused on the heat-up phase of the LOCA, the cooling-down phase should also be considered, as cracking patterns are known to continue evolving during this phase as well.

## Figures and Tables

**Table 1:** Parameter values for the Wiesenack model used in Equation (27) [29].

Parameter	Value
$A_{0}$	0.1148
$A_{1}$	0.0035
$B_{0}$	0.0002474
$B_{1}$	$-8\;\times \;10^{-7}$
*C*	0.0132
*D*	0.00188

**Table 2:** Compiled list of all material properties used in this work.

Property	Symbol	Value	Reference
Fracture Stress	$\sigma_{f}$	200 MPa	[27,35]
Elastic Modulus	*E*	200 GPa	[32]
Poisson’s Ratio	ν	0.33	[32]
Thermal Conductivity	*k*	Halden (Equation (27))	[29]
Density	ρ	10,970 $\mathrm{kg}/m^{3}$	[33]
Specific Heat	$C_{p}$	480 J/kg·K	[34]
Thermal Expansion Coefficient	α	$10^{-5}$ $K^{-1}$	[21]

**Table 3:** Compiled list of all model parameters used in this work.

Parameter	Symbol	Value	Reference
Cohesion Contribution	*A*	6 × $10^{4}$ Pa	This work
Double-Well Contribution	*B*	4 × $10^{4}$ Pa	This work
κ	κ	0.00045 J/m	This work
Surface Energy	γ	1 $J/m^{2}$	[27]
Length Scale	*l*	0.3 mm	This work
Crack Mobility	*L*	1 $\mathrm{MPa}\cdot s^{-1}$	This work

**Table 4:** Summarized results of the pre-existing crack stress test benchmark.

Applied Stress (MPa)	Strain	Strain Energy (Pa)	Crack Growth (Yes/No)
200	0.001	100,000	Yes
100	0.0005	25,000	Yes
90	0.00045	20,250	Yes
80	0.0004	16,000	Yes
70	0.00035	12,250	Yes
67	0.000335	11,222.5	Yes
65	0.000325	10,562.5	No
62	0.00031	9610	No
60	0.0003	9000	No
50	0.00025	6250	No

## Data Availability

The original contributions presented in this study are included in the article. Further inquiries can be directed to the corresponding author.
